# Long-term watermelon continuous cropping leads to drastic shifts in soil bacterial and fungal community composition across gravel mulch fields

**DOI:** 10.1186/s12866-022-02601-2

**Published:** 2022-08-02

**Authors:** Xin Gu, Na Yang, Yan Zhao, Wenhui Liu, Tingfeng Li

**Affiliations:** School of Agriculture, Ningxia University, 750021 Yinchuan, China

**Keywords:** Gravel mulch field, Watermelon continuous cropping, Soil bacteria and fungi, Community composition

## Abstract

Despite the known influence of continuous cropping on soil microorganisms, little is known about the associated difference in the effects of continuous cropping on the community compositions of soil bacteria and fungi. Here, we assessed soil physicochemical property, as well as bacterial and fungal compositions across different years (Uncropped control, 1, 6, 11, 16, and 21 years) and in the watermelon system of a gravel mulch field in the Loess Plateau of China. Our results showed that long-term continuous cropping led to substantial shifts in soil bacterial and fungal compositions. The relative abundances of dominant bacterial and fungal genera (average relative abundance > 1.0%) significantly varied among different continuous cropping years (*P* < 0.05). Structural equation models demonstrated that continuous cropping alter soil bacterial and fungal compositions mainly by causing substantial variations in soil attributes. Variations in soil pH, nutrient, salinity, and moisture content jointly explained 73% and 64% of the variation in soil bacterial and fungal compositions, respectively. Variations in soil moisture content and pH caused by continuous cropping drove the shifts in soil bacterial and fungal compositions, respectively (Mantel *R* = 0.74 and 0.54, *P* < 0.01). Furthermore, the variation in soil bacterial and fungal composition showed significant correlation with watermelon yield reduction (*P* < 0.01). Together, long-term continuous cropping can alter soil microbial composition, and thereby influencing watermelon yield. Our findings are useful for alleviating continuous cropping obstacles and guiding agricultural production.

**Supplementary Information:**

The online version contains supplementary material available at 10.1186/s12866-022-02601-2.

## Introduction

Gravel mulch technology is one of the most crucial methods for surface coverage in dryland regions, which can reduce evaporation and runoff in dry farming areas and thus has become an essential agricultural management measure to promote water-use efficiency in dryland regions [1–3]. Owing to limiting factors including economic interests, tillage and climatic conditions, continuous cropping of watermelon has been commonly conducted in gravel mulch fields for a long time [4, 5]. However, long-term continuous cropping of watermelon may induce soil mineral deficiencies, increase disease incidences [6, 7]. Afterwards, these abiotic and biotic changes can lead to a decrease in watermelon fruit yield and quality [4, 7]. Soil microorganisms play crucial roles in mediating key ecosystem processes and function [8–10]. Hence, the shifts in soil microbial composition can be considered as a sensitive biological indicator for soil health [11]. Previous studies have demonstrated that continuous cropping will significantly alter soil microbial community structure [12–14]. However, due to the difference in cropping systems, planting years and research methodology, no consensus has been reached on the effects of long-term continuous cropping on soil microbial communities [12, 15]. The present study on the effect of watermelon continuous cropping on soil microbial communities in gravel mulch fields is essential for maintaining watermelon fruit yield and quality.

A great number of previous studies have revealed that continuous cropping may induce substantial shifts in soil physicochemical conditions, such as nutrient availability and enzymatic activities, thereby can significantly alter the diversity, abundance and composition of soil microorganisms [12, 13, 16, 17]. As the two most major taxa of soil microorganisms, fungi and bacteria have different dispersal abilities, metabolic activities, and environmental preferences [18–20]. Importantly, soil bacteria and fungi need to compete for similar resources [21]; fungi have a stronger capacity to decompose complex molecules than bacteria [22, 23]. This may lead to the different responses of soil bacterial and fungal communities to the same environmental drivers [24]. Previous studies have reported that different soil physicochemical factors determine soil bacterial and fungal compositions [25, 26]. For example, the community structure of soil bacterial communities is shaped by soil pH [27], but that of soil fungal communities is strongly influenced by soil carbon content in the black soil zone of northeast China [28]. Therefore, soil bacteria and fungi may have different responses to the variation in each soil physicochemical factor caused by long-term continuous cropping. Given that, long-term continuous cropping may have different effects on soil bacteria and fungi. For example, previous studies using the plate culture method have found that soybean continuous cropping would increase the abundance of soil fungi but decrease that of soil bacteria [12]. In past decades, the effect of continuous cropping on soil microbial communities have been well explored in barley system [16], soybean system [12], cucumber system [29], peanut system [30], and cotton system [31]. However, little is still known about how long-term continuous cropping of watermelon interacts with soil physicochemical factors to influence soil bacteria and fungi in gravel mulch fields.

A major objective of our study was to test how long-term continuous cropping and soil physicochemical factors jointly alter soil bacterial and fungal composition, and explore the links between variation in soil microbial composition and watermelon yield. In this analysis, we assessed the soil physicochemical properties, as well as bacterial and fungal compositions under different continuous years (CK, 1, 6, 11, 16, and 21 years) in the watermelon (*Citrullus lanatus*) systems of a gravel mulch field in the Loess Plateau of China. Hence, we attempted to address the following specific questions: (1) Do soil bacterial and fungal composition significantly differ among different continuous years? (2) How do continuous cropping and physicochemical factors jointly drive the variations in soil bacterial and fungal compositions? (3) Do the variation in soil bacterial and fungal composition have significant relationships with watermelon yield?

## Materials and methods

### Site description and sampling

This study was conducted on a gravel mulch cropland in Zhongwei City of Ningxia Hui Autonomous Region (36° 57′ N, 105° 18′ E). As a typical dryland ecosystem, the annual mean precipitation, temperature, and annual mean evaporation of the study region are 247.4 mm, 7.1 ℃, and 2100–3200 mm, respectively. The zonal soil type is mostly ash-calcium, and the zonal vegetation is desert grassland.

Watermelon (Jincheng V) continuous cropping for 1, 6, 11, 16, and 21 years (denoted as 1a, 2a, 11a, 16a, and 21a, respectively) was selected in this study. These cropped systems were managed under same-level nutrients input and field management activities. Additionally, a non-cropped control treatment (CK) was also selected. In total, 18 bulk soil samples (six treatments × three replicates) were collected at the flowering stage of watermelon in 2019. In each treatment, 10 soil cores (20 cm depth) were randomly collected within an area of approximately 100 m^2^ and then mixed thoroughly to form a composite sample (a replicate). The composite soil sample was sieved by a 2 mm mesh and then subdivided into two parts: one portion was stored in thermal insulated boxes (at 4 °C) for determining soil physicochemical properties, and the other portion was stored at − 20 °C for DNA extraction.

### Soil physicochemical property

The contents of soil organic matter (SOM) and total nitrogen (STN) were assessed by K_2_Cr_2_O_7_ oxidation method [32] and Kjeldahl procedure [33], respectively. Soil available nitrogen (SAN) and total phosphorus (STP) contents were determined by alkali diffusion method [34] and molybdenum blue method [35], respectively. Soil available potassium (SAK) extracted with 1 mol/ L ammonium acetate (NH4OAc) was measured be inductively coupled plasma-atomic emission spectrometry [36]. Soil pH was determined using a pH meter with a 1:2.5 ratio of fresh soil to deionized water. Soil moisture content (SM) was measured gravimetrically a er drying soil in an oven at 105 °C for 48 h. Soil available phosphorus (SAP) by the Olsen’s method [37]. Soil water-soluble salinity content (SSC), was determined by using an electric conductometer [38].

### Molecular analyses

Genomic DNA was extracted from 0.5 g fresh soil samples using E.Z.N.A. Soil DNA Kits (OMEGA, United States) following the manufacturer’s instructions. The V3–V4 hypervariable region of the bacterial 16 S rRNA gene was amplified using primers 338 F (5′-ACTCCTACGGGAGGCAGCAG-3′) and 806R (5′-GGACTACNNGGG TATCTAAT-3′) [39]. Universal primers ITS1F (5′-CTTGGTCATTTAGAGGAAGTAA-3′) and ITS2R (5′-TGCGTTCTTCATCGATGC-3′) [40] were used to amplify fungal internal transcribed spacer (ITS) region. These primers contained a set of 8-nucleotide barcode sequences unique to each sample. The PCR program was as follows: 95 °C for 5 min, 25 cycles at 95 °C for 30 s, 55 °C for 30 s, and 72 °C for 30 s with a final extension of 72 °C for 10 min. PCR reactions were performed in triplicate 25 µL mixture containing 2.5 µL of 10 × Pyrobest Buffer, 2 µL of 2.5 mM dNTPs, 1 µL of each primer (10 µM), 0.4 U of Pyrobest DNA Polymerase (TaKaRa), and 15 ng of template DNA. Amplicons were extracted from 2% agarose gels and purified using the AxyPrep DNA Gel Extraction Kit (Axygen Biosciences, Union City, CA, U.S.) according to the manufacturer’s instructions and quantified using QuantiFluor™ -ST (Promega, U.S.). Purified amplicons were pooled in equimolar amounts and paired-end sequenced (2 × 300) on an Illumina MiSeq platform according to the standard protocols.

Fungal and bacterial sequences > 200 bp with an average quality score > 20 and without ambiguous base calls were processed using QIIME packages [41]. These high-quality sequences were clustered into operational taxonomic units (OTUs) based on a 97% similarity threshold using UPARSE [42]. Fungal and bacterial taxonomies were assessed against Silva v128 and UNITE v8.0, respectively [43]. A randomly selected subset of 15,777 and 38,564 bacterial and fungal sequences per sample were used in the subsequent analysis to reduce the effects of different sequencing depths on the analyses. The soil bacterial and fungal raw sequence data used in this study have been submitted in the NCBI Sequence Read Archive under BioProject PRJNA775053.

### Data analysis

Eight soil variables (SOM, STN, STP, SAP, SAN, SAK, SM, SSC, and pH) were used in our analysis. All explanatory variables were standardized to interpret parameter estimates on a comparable scale. Principal component analysis (PCA) was conducted within “vegan” package to reduce soil nutrient data (e.g., SOM, STN, STP, SAP, and SA, SAK) redundancy. The first two soil principal components (SPCs; i.e., SPC1 and SPC2) jointly explained more than 95% of the total variation, and thus were used in the following analysis (Table S1). One-way ANOVA with Tukey’s test was carried out to test the influence of continuous cropping on soil physicochemical conditions. To test the differences in bacterial and fungal taxonomic composition across different continuous years, this study chose the most 8 dominant bacterial genera and 13 dominant fungal genera based on the taxonomic abundance data (average relative abundance > 10% across all samples). One-Way ANOVA was then conducted to assess the significance of group differences among different treatments.

Pairwise Bray–Curtis distance for bacterial and fungal communities and standardized environmental Euclidean distance were calculated within “vegan” package. Permutational analysis of variance (PERMANOVA) was carried out to test the influence of continuous cropping on bacterial and fungal community compositions. Principal coordinate analysis (PCoA) was used to exhibit the variations in bacterial and fungal compositions across different periods. Both PERMANOVA and PCoA were conducted within “vegan” package in R.

Mantel tests (10,000 permutations) were conducted in this study to examine the relationships between soil variables and soil microbial composition. St, structural equation models (SEMs) were constructed to explore the direct and indirect influence of continuous cropping and soil condition on the variation in soil fungal and bacterial composition. Here, direct influence means that given variables can directly alter the community composition of bacteria and fungi, while indirect influence indicates that given variables indirectly alter soil bacterial and fungal compositions via affecting other variables. χ^2^ test, comparative fit index (CFI), goodness of fit index (GFI), and root square mean error of approximation (RMSEA) were used to test whether the models were fitted [44]. Standardized direct and indirect effects were added to evaluate the standardized total effects of each variable. SEM was performed within “lavaan” package.

## Results

### Effects of continuous cropping on soil physicochemical properties and watermelon yield

**Fig. 1:**
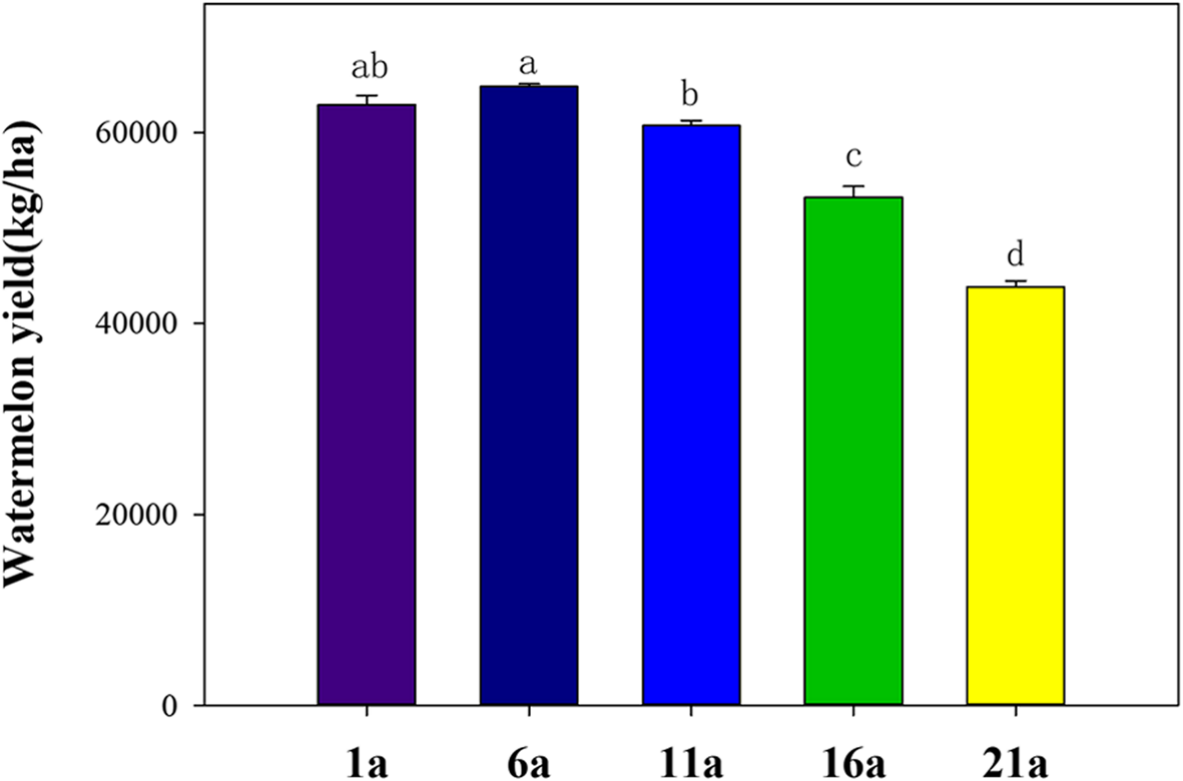
The difference in watermelon yield among different continuous cropping years. Different lowercase letters indicate a significant difference between two treatments

The ANOVA results showed that all soil physicochemical variables significantly varied among the different continuous cropping years (*P* < 0.001, Table 1). Long-time continuous cropping remarkably decreased the STN, STP, SAN, SAK and SAP and remarkably increased SM. SOM initially increased and then decreased with increasing continuous cropping time. By contrast, SSC initially decreased and then increased with increasing continuous cropping time. Soil pH was the lowest in 1a and the highest in 21a. One-Way ANOVA also revealed that watermelon yield was significantly different among different continuous years, and watermelon yield sharply decreased with increasing continuous years (Fig. 1). Therefore, watermelon continuous cropping for 1, 6, 11, 16 and 21 years was selected in this study.

**Table 1:** Difference in soil physicochemical property (mean ± SE) between different continuous cropping years

Variables	CK	1a	6a	11a	16a	21a
SOM (g/kg)	2.79 ± 0.31a	4.75 ± 0.18b	5.58 ± 0.31c	3.31 ± 0.18a	2.69 ± 0.18a	1.24 ± 0.31d
STN (g/kg)	0.48 ± 0.01a	0.46 ± 0.01a	0.4 ± 0.01b	0.36 ± 0.01c	0.33 ± 0.01d	0.3 ± 0.01e
STP (g/kg)	0.81 ± 0.01a	0.75 ± 0.03a	0.56 ± 0.05b	0.48 ± 0.06bc	0.4 ± 0.04 cd	0.31 ± 0.02d
SAN (mg/kg)	24.28 ± 0.82ab	25.69 ± 0.41b	22.62 ± 0.71a	20.27 ± 0.4c	19.8 ± 0.71 cd	17.91 ± 1.08d
SAP (mg/kg)	9.88 ± 0.62a	10.6 ± 1.07a	7.63 ± 0.41b	6.55 ± 0.54b	4.65 ± 0.9c	3.34 ± 0.21c
SAK (mg/kg)	235.67 ± 3.06a	203.53 ± 11.03b	179.05 ± 9.55bc	154.56 ± 4.04 cd	133.14 ± 7.01d	78.04 ± 014.03e
SSC (g/kg)	1.39 ± 0.05a	0.68 ± 0.03b	0.32 ± 0.01c	0.36 ± 0.03c	0.82 ± 0.02d	0.93 ± 0.02e
pH	8.21 ± 0.02a	7.98 ± 0.02b	8.44 ± 0.1c	8.51 ± 0.03 cd	8.6 ± 0.04d	8.74 ± 0.04e
SM (%)	5.22 ± 0.26a	8.28 ± 0.21b	9.7 ± 0.25c	10.56 ± 0.46d	11.34 ± 0.33d	11.26 ± 0.21d

Different lowercase letters indicate a significant difference between two treatments (*P* < 0.05). *SOM *soil organic matter, *STN *soil total nitrogen, *STP *soil total phosphorus, *SAN *soil available nitrogen, *SAP* soil available phosphorus, *SAK *soil available potassium, *SSC *soil water-soluble salinity content, *SM *soil moisture content

### Effects of watermelon continuous cropping on soil bacterial and fungal compositions

**Fig. 2:**
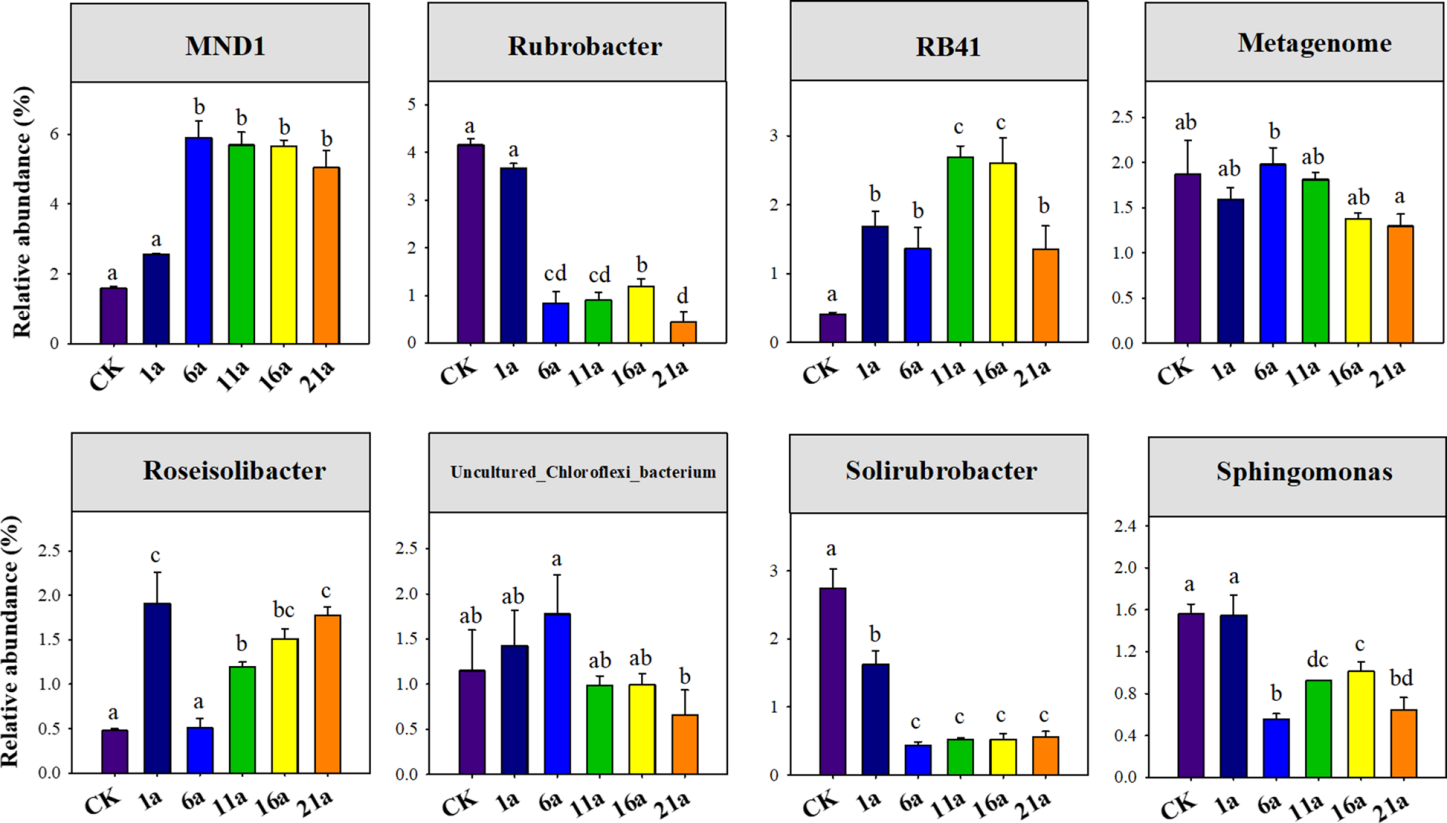
The relative abundance of dominant genera of soil bacteria across different continuous cropping years

**Fig. 3:**
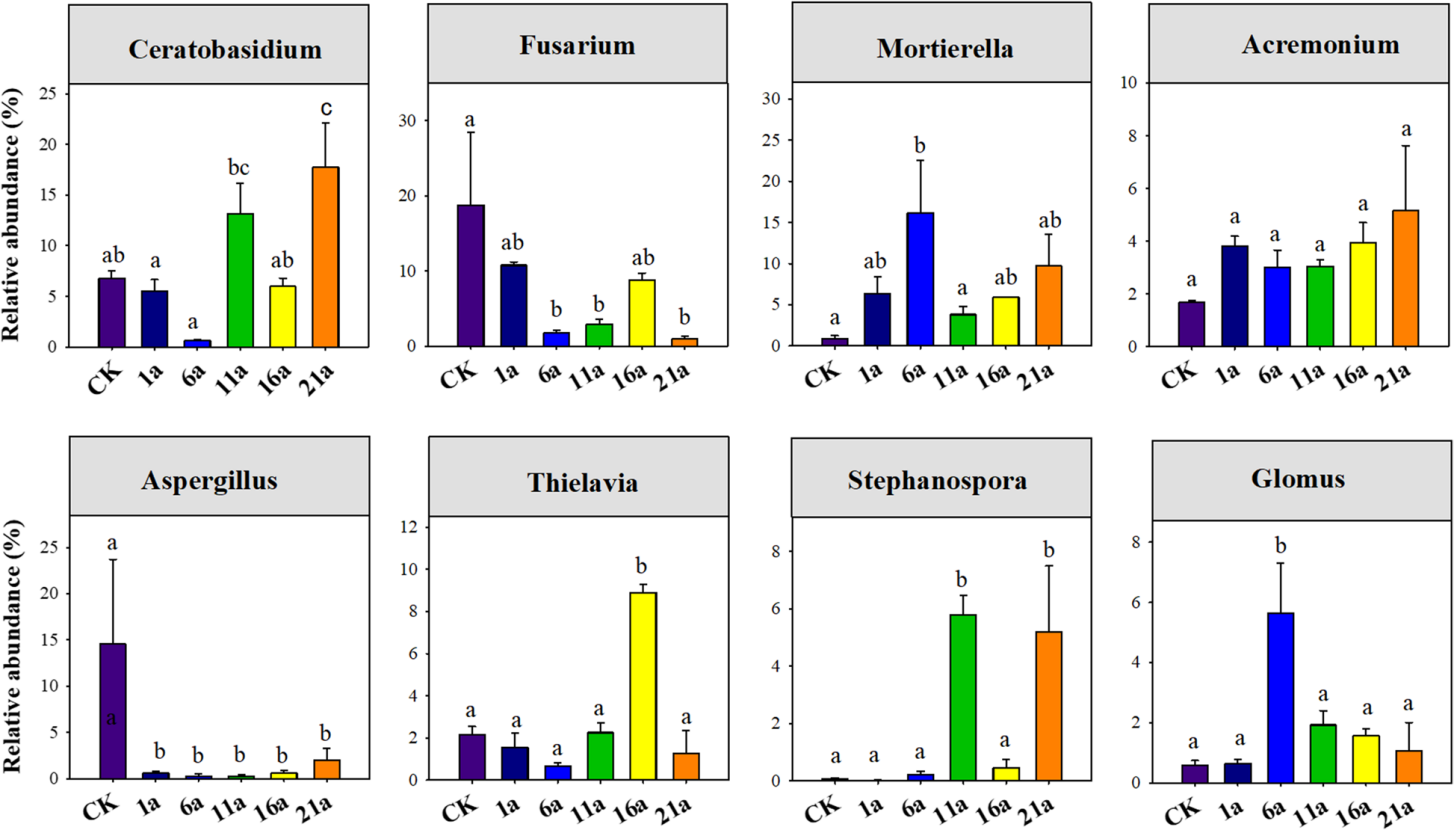
The relative abundance of dominant genera of soil fungi across different continuous cropping years

A total of 256,115 and 687,385 high-quality bacterial and fungal sequences were identified across six treatments, respectively, and classified into 6,435 and 1,349 bacterial and fungal OTUs, respectively. Across all samples, the dominant genera (average relative abundance > 1.0%) of soil bacterial communities were *MND* (4.41%), *Rubrobacter* (1.87%), RB41(1.69%), *Metagenome* (1.66%), *Roseisolibacter* (1.23%), *uncultured_Chloroflexi_bacterium* (1.17%), *Solirubrobacter* (1.08%) and *Sphingomonas* (1.04%). Soil fungal communities were dominated by *Ceratobasidium* (8.32%), *Fusarium* (7.36%), *Mortierella* (7.16%), *Acremonium* (3.45%), *Aspergillus* (3.09%), *Thielavia* (2.81%), *Stephanospora* (1.97%), Glomus (1.92%), *Podospora* (1.83%), *Stachybotrys* (1.76%), *Ramicandelaber* (1.58%), *Conocybe* (1.18%), and *Metarhizium* (1.10%). ANOVA results further showed that the relative abundances of all genera for soil bacteria varied among different continuous cropping years (*P* < 0.001, Fig. 2). Except for *Acremonium*, *Glomus* and *Conocybe*, other 10 dominant fungal genera varied among different continuous cropping years (*P* < 0.001, Fig. 3, Fig. S1).

**Fig. 4:**
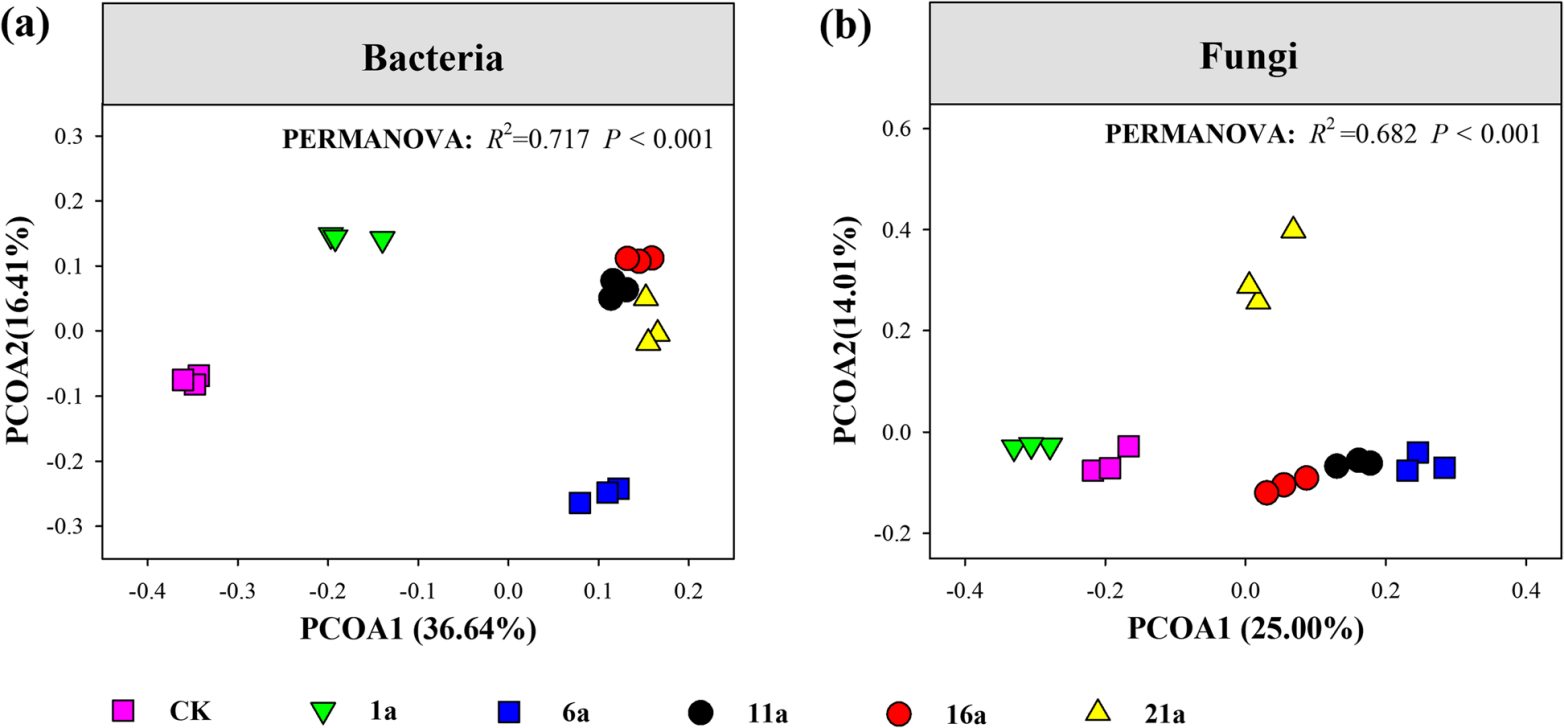
PCoA of soil bacterial (**a**) and fungal (**b**) communities and their variation partition analysis across different continuous cropping periods

PERMANOVA demonstrated that at the OTU level, the species compositions of soil bacteria and fungi significantly differed among different continuous cropping years (*R*^2^ = 0.717 and 0.682, respectively; *P* < 0.001; Fig. 4). Across 6,435 bacterial OTUs, five continuous cropping years only shared 1,347 OTUs. The unique bacterial OTUs detected in a single treatment was 702 for 1a, 327 for 6a, 235 for 11a, 255 for 16a, and 311 for 21a (Fig. S2). Across 1,349 fungal OTUs, five continuous cropping years only shared 348 OTUs. The unique bacterial OTUs detected in a single treatment was 145 for 1a, 48 for 6a, 67 for 11a, 46 for 16a, and 16 for 21a (Fig. S3). These results indicate that different bacterial and fungal species inhabit the soil under different continuous cropping years. Additionally, we observed that the community composition of soil bacteria and fungi exhibited differentially gradual shifts along continuous cropping duration gradients (from CK, 1a to 16a and 21a).

**Fig. 5:**
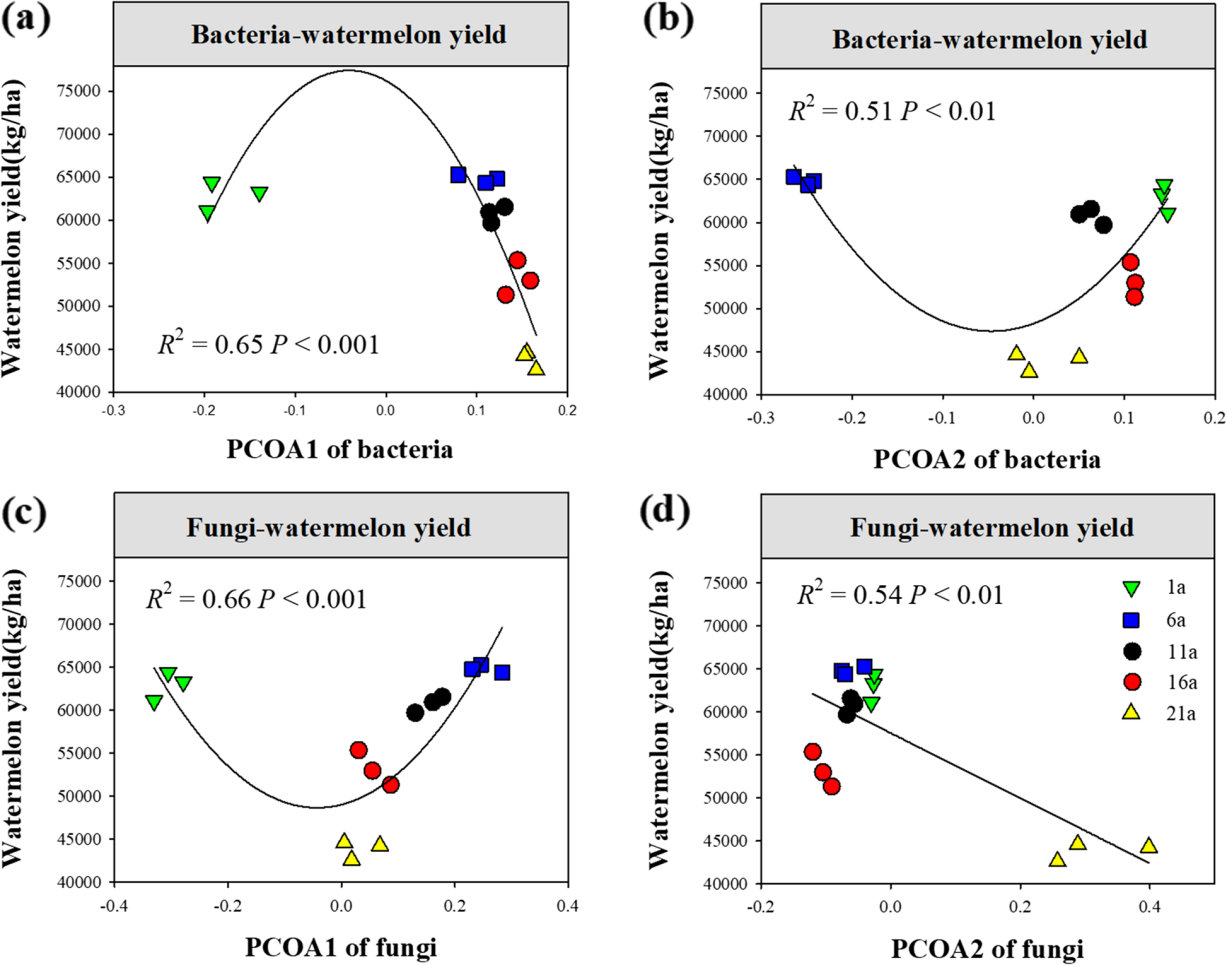
The relationships between watermelon yield and the variation in species compositions of soil bacteria (**a**-**b**) and fungi (**c**-**d**). PCOA1 and PCOA2 mean the first and second axis values of PCoA analysis for soil bacteria and fungi

More importantly, we also observed significant relationships between watermelon yield and the variation in species compositions of soil bacteria and fungi (Fig. 5). For bacteria, the relative abundance of metagenome, Roseisolibacte and Chloroflexi_bacterium had a remarkable correlation with watermelon yield (*P* < 0.05, Table S2). For fungi, the relative abundance of Ceratobasidium, Stephanospora, Podospora and Conocybe had a remarkable correlation with watermelon yield (*P* < 0.05, Table S3).

### Direct and indirect influences of continuous cropping and soil attributes on variations in soil bacterial and fungal compositions

Mantel tests showed that the compositional dissimilarities of soil bacterial and fungal communities were significantly related to the variations in soil nutrient, SSC, pH, and SM (all *P* < 0.01, Table 2). Furthermore, we also found that soil bacterial compositional dissimilarity was more strongly related to the difference in SM (Mantel *R* = 0.75), whereas soil fungal compositional dissimilarity had stronger correlation with pH variation (Mantel *R* = 0.54). Notably, the compositional dissimilarities of soil bacteria and fungi were significantly related to differences in continuous cropping (Mantel *R* = 0.57 and 0.51, respectively).

**Table 2:** Correlations between continuous cropping, soil properties and the species composition of bacteria and fungi

Variables	Bacteria		Fungi	
	Mantel R	P	Mantel R	P
SPC1	0.56	*P* < 0.001	0.54	*P* < 0.001
SPC2	0.49	*P* < 0.001	0.45	*P* < 0.001
SSC	0.56	*P* < 0.01	0.36	*P* < 0.01
pH	0.55	*P* < 0.01	0.54	*P* < 0.01
SM	0.75	*P* < 0.01	0.36	*P* < 0.01
Crop	0.57	*P* < 0.01	0.51	*P* < 0.01

SPC1 and SPC2, the first two soil principal components; *SM *soil moisture content, *Crop *continuous cropping years

**Fig. 6:**
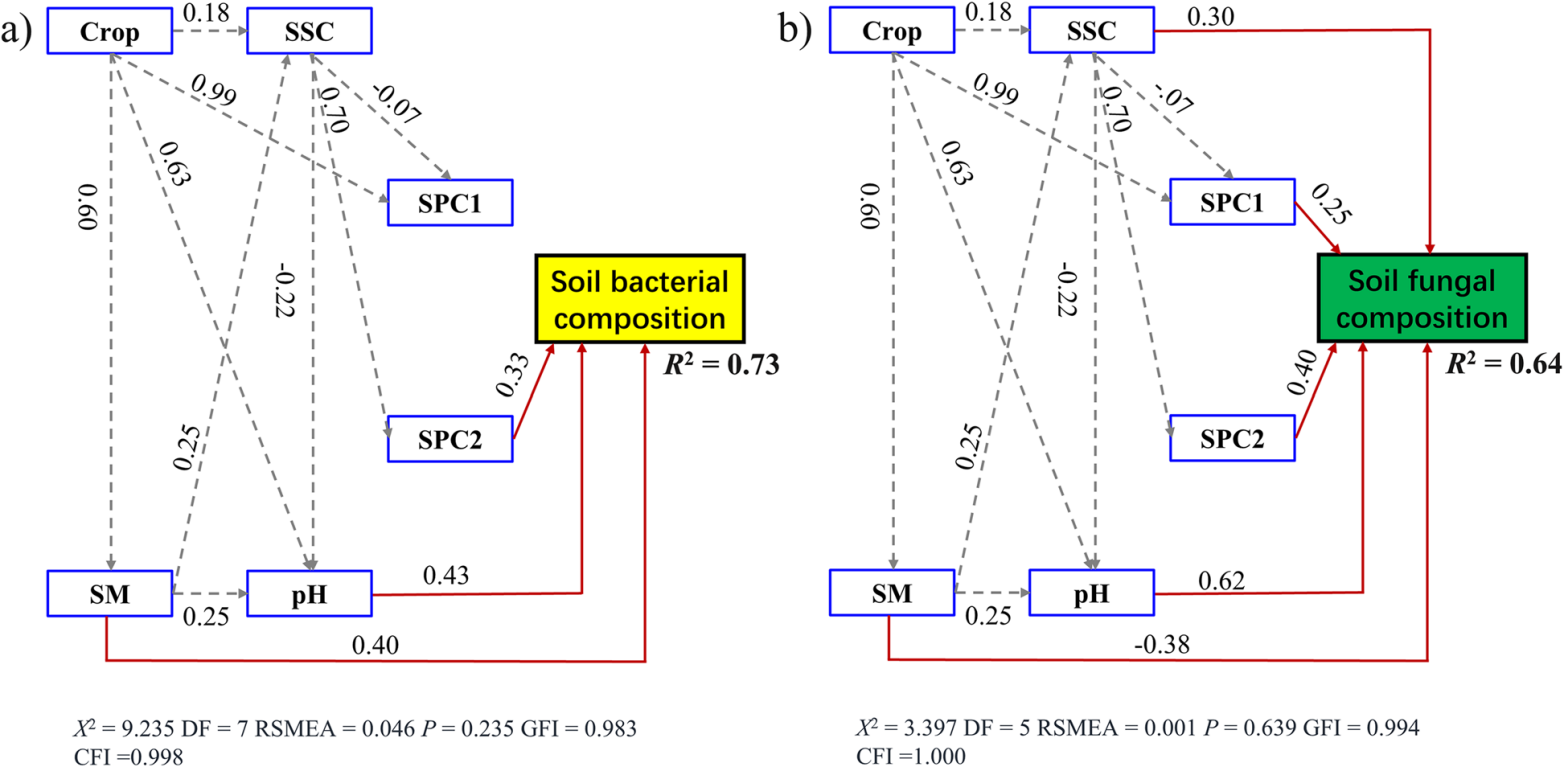
SEM describing the direct and indirect impacts of continuous cropping and soil attributes on species compositions of soil bacterial (**a**) and fungal (**b**) communities. Solid red arrows represent the significant direct paths (*P* < 0.05), while dashed grey arrows indicate the significant indirect paths (*P* < 0.05)

Fitted SEM further confirmed that soil nutrient, SM, pH, SSC, and continuous cropping jointly explained 73% and 64% of the total variations in soil bacterial and fungal community compositions (Fig. 6a and b). Continuous cropping had no direct influence on soil bacterial and fungal communities but can indirectly alter their community composition by affecting soil physicochemical conditions. Additionally. SSC, SM, pH, and soil nutrient had remarkable direct influences on soil bacterial and fungal community compositions.

**Fig. 7:**
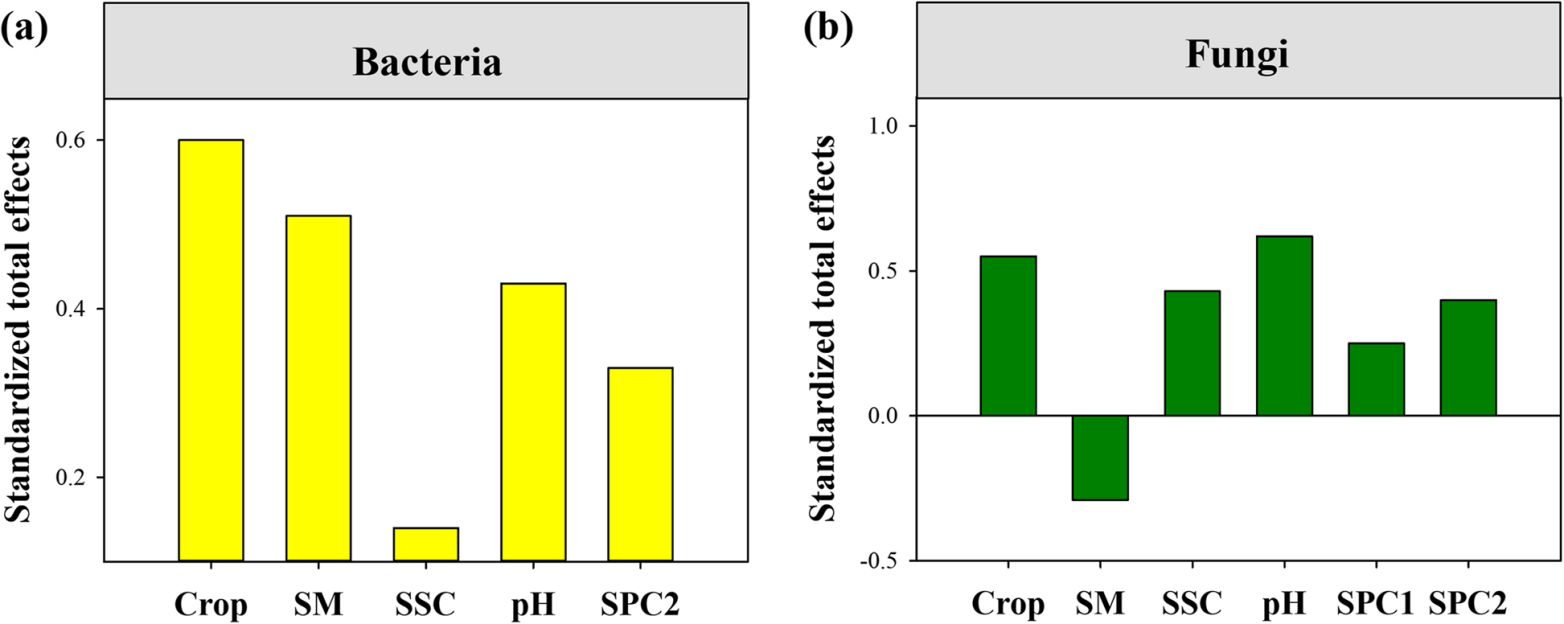
The standardized total influence (direct plus indirect influence) of continuous cropping and soil attributes that derived from the structural equation models of bacterial and fungal community composition

The standardized total effects derived from the SEM revealed that variation in soil bacterial community composition was predominantly driven by continuous cropping, followed by SM, pH, SPC2, and SSC, whereas soil fungal community composition was regulated by pH, continuous cropping, SPC2, SSC, SM and SPC1 (Fig. 7).

## Discussion

### Long-term continuous cropping alters soil physicochemical properties

The long-term continuous cropping of a single crop can induce agricultural ecosystem degeneration, including crop yield reduction, nutrient imbalance, and deterioration of soil physicochemical properties [13, 45]. However, no consensus has been reached on the impact of continuous cropping on soil physicochemical properties across different crop systems [12]. In this study, we observed that soil nutrient, pH, salinity, and moisture content considerably varied across the different continuous cropping years. Different from previous reports that long-term continuous cropping can improve soil nutrient contents [12, 14, 46], we found that soil nutrient contents obviously declined with the increase in continuous cropping duration. A previous study reported that the continuous cropping of cropped banana increases soil pH [46]. By contrast, we found that soil pH decreased in la and then increased with the increase in the duration of watermelon continuous cropping. Liu et al. [12] and Zhong et al. [46] observed that soil organic carbon is high in continuous cropping treatment, whereas our results showed that SOM increased in short-term treatments (la and 6a) and then decreased in long-term continuous cropping (11a, 16a, and 21a). In addition, soil moisture content substantially increased in the continuous cropping treatment, which may be because long-term irrigation and crop cover decreased soil water evaporation (Table 1). Together, these findings suggest that watermelon continuous cropping will substantially alter the soil conditions in a gravel mulch field, but its impact on different soil physicochemical properties varies remarkably.

### Long-term continuous cropping alters soil bacterial and fungal compositions by affecting soil physicochemical properties

A large number of previous studies have reported that long-term continuous cropping significantly changes soil microbial composition [16, 17, 45]. In agreement with previous studies [5], our results demonstrated that bacterial and fungal community compositions varied considerably among different durations of watermelon continuous cropping. We also observed significant variation in dominant bacterial and fungal genera along the gradient of continuous cropping year, which is consistent with previous findings [17, 47]. However, we also found different responses of fungal and bacterial genera to continuous cropping years. For instance, the relative abundance of bacterial *MND*1 and fungal *Ceratobasidium* increased with increasing continuous cropping years, while that of bacterial *Rubrobacter* and *Solirubrobacter*, and fungal *Fusarium* showed opposite trends, suggesting that although long-term continuous cropping will alter soil microbial composition, its effect varies between microbial taxa.

Interestingly, soil bacterial composition was more strongly altered by continuous cropping rather than soil fungal composition. Long-term continuous cropping gradually reduced soil nutrient contents and altered soil pH, organic carbon and salinity. Functional traits could mediate species fitness and performance [48, 49]. Soil bacteria and fungi need to compete for similar resources; fungi have stronger capacity to decompose complex molecules than bacteria [22, 23]. Moreover, soil fungi can maintain community stability by generating multiple mutualism (e.g., mycorrhizae and rhizobia) with crops [50–52]. Hence, soil fungi may have greater tolerance and adaptability to the variation in soil physicochemical properties than bacteria. As a result, continuous cropping can have a strong effect on soil bacterial composition. Additionally, we observed different shifts in soil bacterial and fungal compositions across different durations of continuous cropping. This result is partly because the major soil factors that drive soil bacterial and fungal compositions changed differently across different durations of continuous cropping.

SEM revealed that long-term continuous cropping had no direct influence on soil bacterial and fungal compositions, it could alter soil bacterial and fungal species compositions by affecting soil conditions. Soil factors, such as soil pH and nutrient, are the major factors that drive soil microbial community composition [53–55]. However, the relative influence of different soil factors on microbial composition differed among bacterial and fungal communities [27, 28]. Mantel test and SEM together confirmed that variation in soil bacterial composition mainly driven by soil moisture content, followed by soil pH, nutrient, and salinity. By contrast, soil fungal composition was controlled by soil pH, followed by nutrient, salinity, and moisture content. Expectedly, soil moisture content determined the community composition of soil bacteria because water availability drives biodiversity and ecosystem functioning [56, 57]. Notably, we found that soil bacterial composition was more influenced by soil moisture content than the soil fungal. This may be due to the fact that fungal hyphae facilitate access to soil water [58], and their chitinous cell walls increase their resistance to the variation in soil moisture content [59]. Moreover, soil pH also influences soil microbial assembly [55, 60]. Soil pH is a key determinant of soil fungal community composition [61]. Therefore, soil pH plays an important role in shaping soil fungal composition in gravel mulch field. We also observed that soil pH and moisture content influence bacterial and fungal compositions, respectively. Our result was partly consistent with traditional viewpoints [55, 62]. Additionally, soil salinity is considered the key driver of soil microbial communities [38]. Our findings also showed that soil salinity content plays a role in altering soil bacterial and fungal compositions. These results indicate that soil fungi and bacteria have different responses to the variation in each soil factor caused by long-term continuous cropping. Together, our study provides empirical evidence that long-term continuous cropping of watermelon alters soil bacterial and fungal compositions mainly by affecting soil physicochemical properties.

### Changing community composition of soil bacteria and fungi leads to a decline in watermelon yield

Numerous studies have reported that long-term continuous cropping leads to alterations in soil microbial composition, and crop yield reduction [17, 31, 46]. However, little is known about the influence of soil microbial changes on crop yield reduction. In this study, we observed significant relationships between watermelon yield reduction and the variation in OTU-level compositions of soil bacteria and fungi. More importantly, we also found that the relative abundance of bacterial Metagenome was positive related to watermelon yield. An increase in the relative abundance of fungal *Ceratobasidium* and *Stephanospo*ra, and a decrease in that of *Podospora* led to watermelon yield reduction, indicating that long-term continuous cropping may decrease watermelon yield by changing soil microbial composition, especially by disturbing the balance between beneficial and pernicious microorganisms [4]. In this study, we only analysed the taxonomic composition of soil bacteria and fungi. Future research should explore the key functional taxa that improve soil quality and increase watermelon yield through *functional-annotations* and *phylogenomics*, and take the combination of bio-organic fertilizers, crop rotation and functional microorganisms into account to effectively prevent soil degradation and promote crop growth [4, 12, 63].

## Conclusions

This study conducted a comprehensive comparison of the influence of continuous cropping on soil bacterial and fungal compositions and summarized the variations in soil factors caused by continuous cropping that drove shifts in soil bacterial and fungal compositions. Our results observed that the community composition of soil bacteria and fungi were remarkably altered by continuous cropping in gravel mulch field. SEM further demonstrated that continuous cropping indirectly altered soil bacterial and fungal compositions by causing remarkable variations in soil attributes. In addition, soil bacterial and fungal compositions were driven by variations in soil moisture content and pH caused by continuous cropping, respectively. As a result, soil bacterial and fungal communities exhibited differential compositional shifts across different years of continuous cropping. Additionally, the variation in soil bacterial and fungal composition had a significant correlation with watermelon yield reduction. Together, our findings provide first-hand evidence that long-term continuous cropping of watermelon alters soil bacterial and fungal compositions mainly by affecting soil physicochemical properties in gravel mulch fields.

## Supplementary Information

**Additional file 1.**

## Data Availability

All data generated or analyzed during this study are included in this article and its supplementary information files. The soil bacterial and fungal raw sequences used in data analysis have been submitted in the NCBI Sequence Read Archive under BioProject PRJNA775053.
