# Supplementary material for: Long-term watermelon continuous cropping leads to drastic shifts in soil bacterial and fungal community composition across gravel mulch fields
Source: BMC Microbiol. 2022 Aug 2;22:189. doi: 10.1186/s12866-022-02601-2 (PMC9344729; doi:10.1186/s12866-022-02601-2)
Supplement: Supplementary file 1 — Additional file 1. [file 12866_2022_2601_MOESM1_ESM.doc]

**Table S1** Principal component analysis (PCA) of soil nutrient data

|  | **SPC1** | **SPC2** | **SPC3** | **SPC4** | **SPC5** | **SPC6** |
| --- | --- | --- | --- | --- | --- | --- |
| SOM | 0.29 | -0.94 | 0.06 | -0.05 | -0.16 | 0.07 |
| STN | 0.43 | 0.18 | -0.02 | 0.10 | -0.58 | -0.66 |
| STP | 0.43 | 0.26 | -0.18 | -0.14 | -0.43 | 0.71 |
| SAN | 0.43 | 0.01 | -0.27 | 0.76 | 0.40 | 0.06 |
| SAP | 0.43 | 0.05 | -0.40 | -0.62 | 0.48 | -0.20 |
| SAK | 0.42 | 0.15 | 0.85 | -0.07 | 0.25 | 0.05 |
| Eigenvalue | 2.26 | 0.80 | 0.34 | 0.28 | 0.17 | 0.14 |
| Proportion explained (%) | 85.33 | 10.57 | 1.95 | 1.35 | 0.49 | 0.32 |
| Cumulative proportions (%) | 85.33 | 95.90 | 97.85 | 99.19 | 99.69 | 100.00 |

**Table S2** Relationships between watermelon yield and the variation in relative abundance of dominant bacterial genera

|  | MND1 | Rubrobacter | RB41 | Metagenome | Roseisolibacter | Uncultured_Chloroflexi_bacterium | Solirubrobacter | Sphingomonas |
| --- | --- | --- | --- | --- | --- | --- | --- | --- |
| Yield | -0.01 | 0.39 | -0.04 | 0.775^**^ | -0.621^*^ | 0.532^*^ | -0.10 | 0.09 |

Spearman’s rank correlation tests were used. * *P* < 0.05, ** *P* < 0.01

**Table S3** Relationships between watermelon yield and the variation in relative abundance of dominant fungal genera

|  | Ceratobasidium | Fusarium | Mortierella | Acremonium | Aspergillus | Thielavia | Stephanospora | Glomus | Podospora | Stachybotrys | Ramicandelaber | Conocybe | Metarhizium |
| --- | --- | --- | --- | --- | --- | --- | --- | --- | --- | --- | --- | --- | --- |
| Yield | -0.654^**^ | 0.196 | 0.114 | -0.193 | -0.489 | -0.250 | -0.568^*^ | 0.418 | 0.593^*^ | 0.043 | -0.096 | 0.736^**^ | -0.064 |

Spearman’s rank correlation tests were used. * *P* < 0.05, ** *P* < 0.01


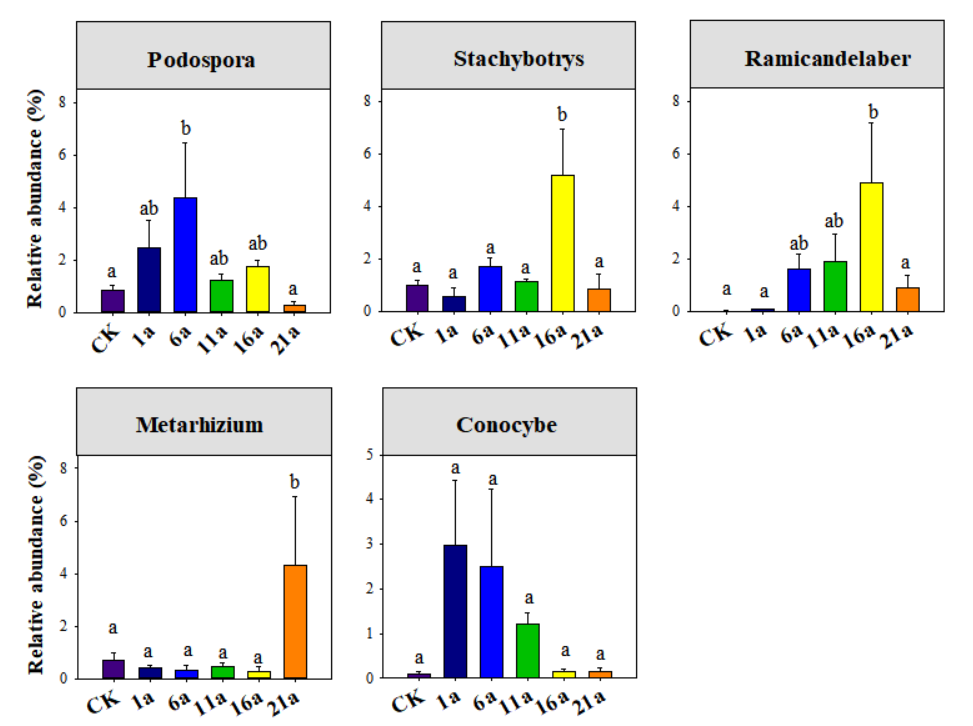


**Figure S1** The relative abundance of dominant genera of soil fungi across different continuous cropping periods

Fi
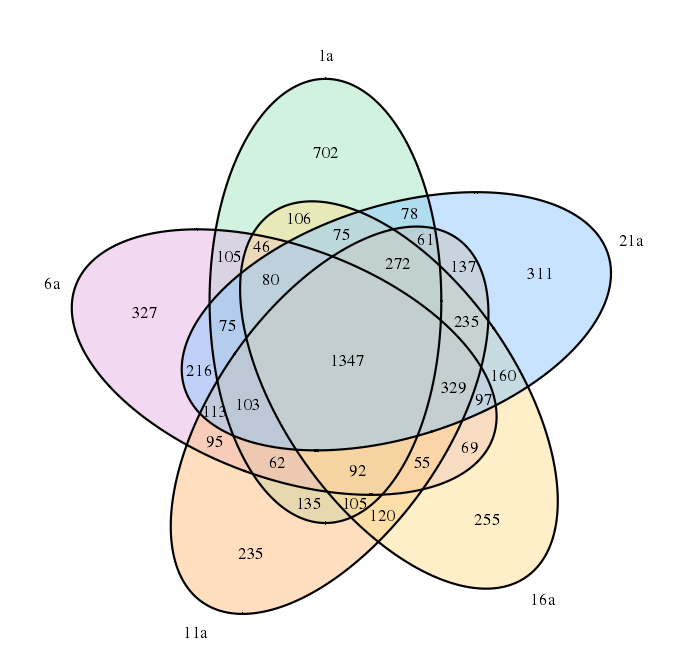


**Figure S2** Venn diagram showing the number of unique and overlapping bacteria OTUs different continuous cropping years


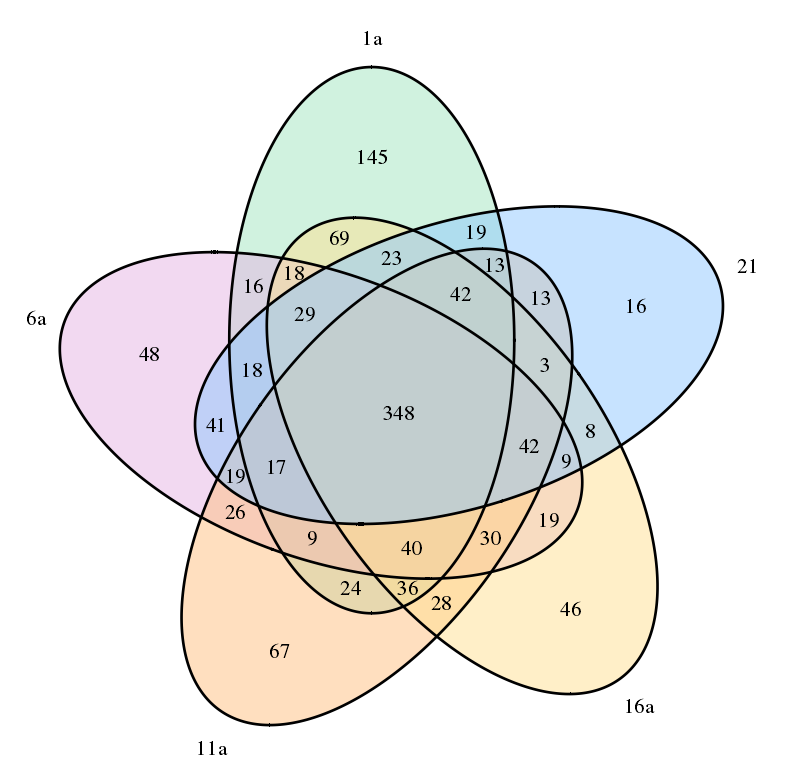


**Figure S3** Venn diagram showing the number of unique and overlapping bacteria OTUs different continuous cropping years
